# Louisiana State University

**Published:** 1897-09

**Authors:** 


### LOUISIANA STATE UNIVERSITY.

The announcement for 1897–'98 of the Louisiana State University and Agricultural and Mechanical College at Baton Rouge informs us of the return of Dr. W. H. Dalrymple to the chair of veterinary science. We note the existence of a commodious veterinary hospital in connection with the school, and that veterinary science is a part of the curriculum of the junior and senior classes in agriculture, embracing the anatomy, physiology, and pathology of the different systems of equines and bovines as well as diseases, obstetrics, orthopedic surgery, dermatology, and helminthology.

